# Genetically supported causality between gut microbiota and frailty: a two-sample Mendelian randomization study

**DOI:** 10.3389/fmicb.2024.1324209

**Published:** 2024-04-17

**Authors:** Zi Wang, Shuai Han, Yinggang Xiao, Yang Zhang, Yali Ge, Xin Liu, Ju Gao

**Affiliations:** ^1^Yangzhou University Medical College, Yangzhou, China; ^2^Department of Anesthesiology, Institute of Anesthesia, Emergency and Critical Care, Yangzhou University Affiliated Northern Jiangsu People’s Hospital, Yangzhou, China

**Keywords:** gut microbiota, frailty, Mendelian randomization, older people, causal association, genome-wide association study

## Abstract

**Background:**

A mounting body of evidence suggests a strong connection between gut microbiota and the risk of frailty. However, the question of causality remains unanswered. In this study, we employed a Mendelian randomization (MR) approach to assess potential causal relationships between gut microbiota and the risk of frailty.

**Materials and methods:**

Summary statistics for the gut microbiome were obtained from a genome wide association study (GWAS) meta-analysis of the MiBioGen consortium (*N* = 18,340). Summary statistics for frailty were obtained from a GWAS meta-analysis, including the UK Biobank and TwinGene (*N* = 175,226). Our primary analysis utilized the inverse variance weighted (IVW) method. To enhance the robustness of our results, we also applied weighted median methods, MR Egger regression, and MR pleiotropy residual sum and outlier test. Finally, we conducted reverse MR analysis to investigate the potential for reverse causality.

**Results:**

IVW method identified 7 bacterial taxa nominally associated with the risk of FI. *Class Bacteroidia* (*p* = 0.033) and *genus Eubacterium ruminantium* group (*p* = 0.028) were protective against FI. In addition, *class Betaproteobacteria* (*p* = 0.042), *genus Allisonella* (*p* = 0.012), *genus Bifidobacterium* (*p* = 0.013), *genus Clostridium innocuum group* (*p* = 0.036) and *genus Eubacterium coprostanoligenes group* (*p* = 0.003) were associated with a higher risk of FI. No pleiotropy or heterogeneity were found.

**Conclusion:**

The MR analysis indicates a causal relationship between specific gut microbiota and FI, offering new insights into the mechanisms underlying FI mediated by gut microbiota.

## 1 Introduction

Frailty is commonly defined as a decline in both physical resilience and cognitive recovery capacity (Mueller et al., 2016), heightening susceptibility to stressors and providing a more accurate prediction of adverse health outcomes such as hospitalization, dependence, and mortality, compared to chronological age alone (Mitnitski et al., 2001). This condition serves as a crucial indicator of overall health deficiencies amidst the global trend of demographic aging. United Nations, Department of Economic and Social Affairs, Population Division (2015) projections anticipate the global population aged 60 and above will reach 1.2 billion by 2025. The prevalence of frailty imposes a significant burden on older adults, their families, and society at large. Numerous meta-analyses indicated that frailty correlates with a heightened risk of all-cause mortality, as well as cause-specific mortality from cardiovascular disease (CVD), cancer, and respiratory illness (Ferrucci and Fabbri, 2018; Kojima et al., 2018; Yang et al., 2018; Peng et al., 2022). Furthermore, frailty escalates healthcare costs for the elderly, leading to catastrophic health expenditures (Chi et al., 2021). However, despite its wide use in clinical practice, there is no consensus on the definition of frailty. The most common approach to measure frailty is the Fried frailty phenotype with biological underpinnings encompassing five components including unintentional weight loss, exhaustion, weakness, slowness, and inactivity (Hoogendijk et al., 2019). In clinical practice, frailty is often identified by reduced physical strength and endurance, sometimes occurring without noticeable cognitive impairments. Recognizing the greater susceptibility of physically frailty individuals to adverse outcomes, Searle et al. (2008) introduced the frailty index (FI) to comprehensively assess this multifaceted condition. The FI is derived from an analysis of the proportion of age-related impairments across 30 distinct physiological parameters.

The burgeoning research on gut microbiota has unveiled its pivotal role in aging and frailty, highlighting how it undergoes dynamic changes correlating with age and health status. This microbiota significantly impacts immune system functionality, essential for warding off age-related diseases (Hasan and Yang, 2019; Rinninella et al., 2019). Although the pathophysiological mechanisms of frailty have not been fully elucidated, current studies identify inflammation as one of the core mechanisms (Soysal et al., 2016). In recent years, a new hypothesis regarding the origin of inflammation in the digestive tract, especially the imbalance of intestinal homeostasis, has attracted the attention of the aca demic community (de Jong et al., 2016). Notably, intestinal inflammation is linked to disruptions in gut microbiota, emphasizing its significance in health and disease (Willing et al., 2010; Chen et al., 2022). The gut, as the primary interface with external microorganisms, plays a key role in managing chronic inflammation associated with frailty. Previous research indicated that an ecological imbalance in the intestine leads to the transformation of gut microbiota into pathogenic bacteria and a decrease in microbial diversity (Gomaa, 2020). This imbalance enhances the permeability of the mucosal barrier, facilitating the entry of bacteria and their by-products into the human body via the intestine, thereby inducing systemic inflammation (Zapata and Quagliarello, 2015; Thevaranjan et al., 2017; Mou et al., 2022). Consequently, it is plausible to suggest a link between the gut microbiota and frailty. Recent investigations have also elucidated potential mechanisms through which gut microbiota may contribute to the aging and frailty processes (Chen et al., 2022). These effects can manifest directly via the gut microbiota itself or its metabolites. A recent narrative review has underscored alterations in the gut microbiota composition among frail patients (Sánchez y Sánchez de la Barquera et al., 2022). Consequently, the gut microbiota appears poised to play a pivotal role in the frailty paradigm.

While the connection between gut microbiota and the frailty syndrome is well-documented, the exact nature of this causal relationship remains uncertain. Mendelian randomization (MR) analysis, a statistical method for discerning potential causation from observed correlations, provides a valuable tool in this context (Thanassoulis, and O’donnell, 2009). MR utilizes genetic variations associated with the exposure of interest as instrumental variables to assess the relationship between these instruments and the outcomes of concern (Emdin et al., 2017). In recent years, MR analysis has been applied to explore the potential causal relationship between gut microbiota and disease risk genes (Liu et al., 2023; Zhang and Zhou, 2023). There is an urgent need to investigate the potential causal connection between gut microbiota and the risk of frailty syndrome.

In this study, we sought to elucidate the potential causal relationship between gut microbiota and the FI. Additionally, we aimed to pinpoint specific pathogenic bacterial classifications contributing to this relationship. To achieve these objectives, we conducted two-sample MR studies utilizing data sourced from the whole genome association study (GWAS).

## 2 Methods

### 2.1 Study design

**Figure 1:**
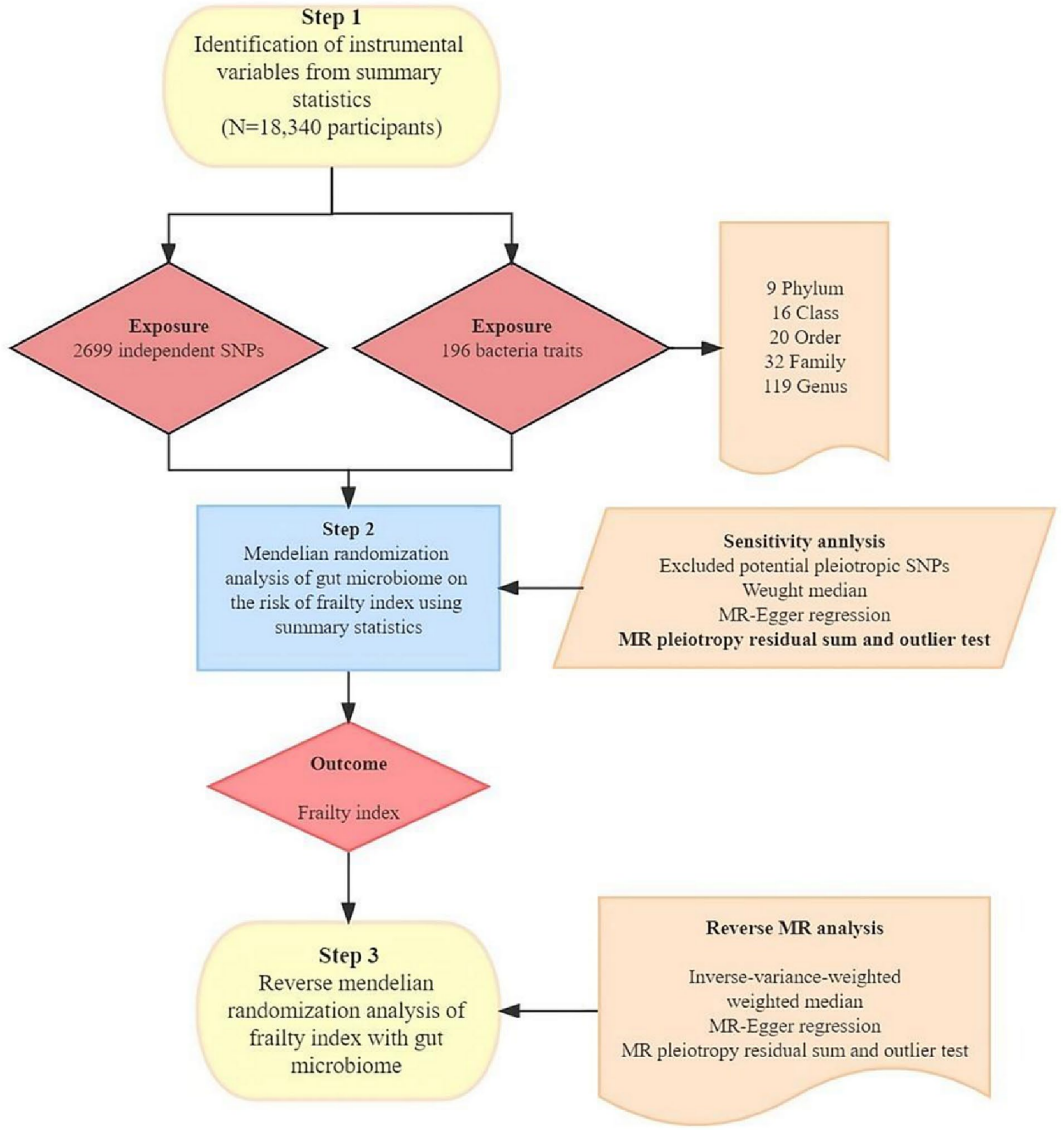
The study design of the associations of gut microbiota and frailty index. MR, Mendelian randomization; SNP, single nucleotide polymorphism.

In our investigation, we examined the association between gut microbiota and the FI through a two-sample MR approach. This method relies on three fundamental assumptions to minimize the effects of confounding variables: (1) the use of single nucleotide polymorphisms (SNPs) significantly related to gut microbiota as instrumental variables (IVs); (2) the requirement that these IVs are independent, i.e., they have no associations with other confounders like age or smoking; and (3) the necessity that the IVs impact the outcome exclusively via the exposure under study, thereby precluding any influence through other pathways, as illustrated in Figure 1.

### 2.2 Ethical review

Data at the summary level utilized for analysis were compiled and sourced from published research studies. These studies adhered to the Declaration of Helsinki guidelines and received authorization from relevant institutional ethics committees, negating the need for further ethical approval. Additionally, this study was conducted in strict accordance with the STROBE-MR guidelines (Skrivankova et al., 2021).

### 2.3 Data sources

The FI serves as a robust tool for frailty assessment, quantifying frailty through the accumulation of deficits across multiple domains such as symptoms, signs, laboratory abnormalities, and disease diagnoses, as listed in Supplementary Table S1. This quantification involves calculating the ratio of observed deficits to the total considered deficits (Mitnitski et al., 2001), with specifics on these deficits detailed in Supplementary Table S2. We derived FI data from a GWAS meta-analysis by Atkins et al. (2021), which included 175,226 participants of European descent. The sample comprised 164,610 UK Biobank participants, aged between 60 and 70 years (mean age of 64.1), with 51.3% females, and 10,616 TwinGene participants, aged between 41 and 87 years (mean age of 58.3), with 52.5% female representation. Utilizing the Rockwood FI, based on the deficit accumulation model, we measured frailty outcomes. Deficits were scored as 0 or 1, with 0 indicating the absence of a deficit. Individual FIs were calculated by dividing the number of deficits by 49, with higher values indicating increased frailty levels. Analysis showed mean deficit proportions of 0.129 ± 0.075 in the UK Biobank cohort and 0.121 ± 0.080 in the TwinGene cohort. The FI has demonstrated strong predictive power for various adverse health outcomes, making it a preferred measure for frailty assessment, especially in younger populations (Blodgett et al., 2015; Theou et al., 2016; Williams et al., 2019).

Genetic proxies for gut microbiota were sourced from the MiBioGen consortium, which executed an extensive genome-wide meta-analysis that integrated human genome-wide genotypes with fecal 16S rRNA sequencing data (Kurilshikov et al., 2021). This elaborate meta-analysis encompassed 18,340 participants across 24 cohorts, primarily of European ancestry. After excluding 15 unclassifiable bacterial taxa, the Mendelian randomization (MR) analysis included 9 phyla, 16 orders, 20 families, 32 genera, and 119 species. The GWAS data for all participating cohorts were adjusted for covariates, including sex, age, genetic principal components, and additional relevant factors.

### 2.4 Instrumental variable selection

We began the analysis by excluding 15 bacterial traits that lacked specific names. This resulted in a dataset consisting of 196 distinct bacterial traits, distributed across 9 phyla, 16 classes, 20 orders, 32 families, and 119 genera. Next, we selected instrumental variables (IVs) using a significance threshold of *p* < 1.0 × 10^−5^ (Sanna et al., 2019). To ensure that the IVs were derived from independent loci, we applied a linkage disequilibrium (LD) threshold of *R*^2^ < 0.001 and a clumping distance of 10,000 kb (Auton et al., 2015). We used the “TwoSampleMR” package on the 1,000 Genomes EUR data. This process retained the SNPs with the most significant *p*-values associated with each trait for clumping alongside the 196 bacterial traits, identifying a total of 2,699 independent SNPs linked to these bacterial traits. In the reverse MR analysis, we applied a stricter significance threshold (*p* < 5 × 10^−8^) for selecting IVs linked to FI, as described in Table 2 of the preceding study (Atkins et al., 2021). We extracted relevant information, including the effect allele, effect size (*β*-value), standard error, and *p*-value for each SNP. To assess the strength of the instruments, we calculated the proportion of variance explained (R^2^) and F-statistics using the following equations: *R*^2^ = 2 × MAF × (1 − MAF) × *β*^2^ and *F* = *R*^2^(*n*−*k*−1)/[*k*(1−*R*^2^)]. Here, “MAF” represents the minor allele frequency of the SNPs used as IVs, “*n*” is the sample size, and “*k*” is the number of IVs employed (Kamat et al., 2019).

To substantiate the second Mendelian randomization assumption, the PhenoScannerV2 database was utilized to investigate each instrumental variable and its associated proxies. Subsequently, SNPs linked to confounders were identified and excluded. Following this, SNPs that serve as surrogate markers for gut microbiota, filtered from the FI GWAS summary data, were isolated. In instances where one or more SNPs were not present in the FI GWAS repository, the corresponding proxy instruments were omitted from analysis.

To check whether estimates of the effect of causality might be affected by weak instrument bias, the strength of IVs was tested using the F statistic. No significant weak instrumental bias is considered to exist if the corresponding F-statistic >10.

### 2.5 Statistical analysis

Leveraging cohort data from initial GWAS, we meticulously ensured the absence of sample overlap between the exposures and outcomes. The cornerstone of our analysis was the two-sample MR approach, strategically selected to unravel the causal dynamics between instrument-exposure and instrument-outcome associations.

Our investigative approach incorporated a spectrum of methods to examine the hypothesized causal relationship between gut microbiota and the frailty index (FI). These methodologies included fixed/random effects inverse variance weighting (IVW), the weighted median approach, MR Egger regression, and the MR pleiotropic residual and outlier (MR-PRESSO) test. The IVW method, acknowledged for its accuracy in effect estimation, was employed as our primary analysis tool, in line with standard practices in MR research (Yavorska and Burgess, 2017; Larsson and Burgess, 2022). To enhance the validity of our MR outcomes, sensitivity analyses were also performed using the Weighted Median and MR-Egger methods. The impact of each SNP on the exposure-outcome was quantified using the Wald ratio method (Pierce and Burgess, 2013), followed by an amalgamation of these estimates through the IVW method for a unified effect estimate. The weighted median method presupposes the validity of most instruments (Bowden et al., 2016), whereas the MR-Egger method detects potential horizontal pleiotropy via a non-zero intercept (Bowden et al., 2015). The MR-PRESSO test, optimally suited for scenarios where horizontal pleiotropy is present in less than half of the instruments, was also implemented to identify any such pleiotropy (Verbanck et al., 2018). The heterogeneity of SNPs, which might affect the outcome through unknown pathways, was evaluated using Cochran’s *Q*-test, *I*^2^ statistics, and leave-one-out analysis. A significant heterogeneity was marked by an *I*^2^ exceeding 25% and a *p*-value less than 0.05 in the Cochran *Q*-test (Greco M et al., 2015). SNPs indicative of pleiotropy or heterogeneity were excluded as per the findings from the MR-PRESSO and leave-one-out analyses, followed by repeated MR analyses for final estimations.

False discovery rate (FDR) correction was conducted by applied q-value procedure, with a false discovery rate of *q*-value <0.1 (Johen and Robert, 2003). Genera of gut microbiota and PE were considered to have a suggestive association when *p* < 0.05 but *q* ≥ 0.1.

For each trait, the addition of one unit was quantified in terms of odds ratio (OR) and its corresponding 95% confidence interval (CI). To address multiple testing, All MR analyses were conducted using R version 4.3.1^1^ with the “Mendelian randomization,” “TwoSampleMR,” and “MR-PRESSO” packages.

## 3 Results

### 3.1 Characteristics of SNPs

We meticulously examined 196 individual bacterial taxa instrumental variables (IVs). Among these, 2,699 IVs exhibited locus-wide significance with a threshold of *p* < 1 × 10^−5^. After mitigating the impact of linkage disequilibrium (LD) within distinct bacterial groups, we pinpointed 2,527 SNPs linked to FI. For a comprehensive list of the selected IVs, please refer to Supplementary Table S3. Notably, the *F*-statistics associated with these IVs consistently surpassed 10, signifying a low susceptibility to weak instrumental bias.

### 3.2 Causal effect of gut microbiota on FI

**Figure 2:**
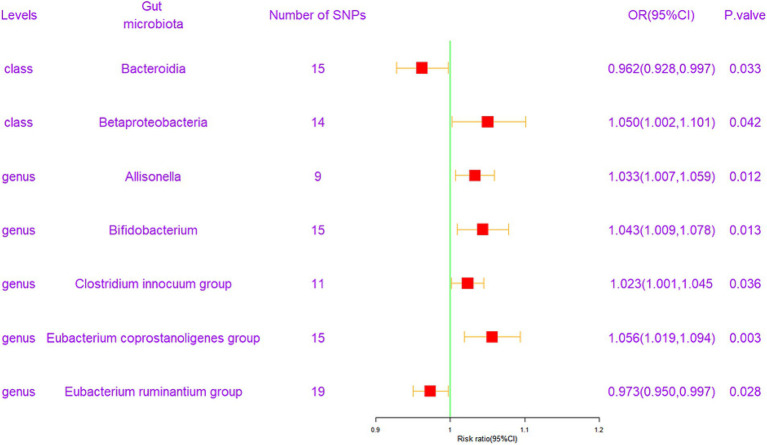
Forest plot of the associations between genetically determined 7 bacterial traits with the risk of frailty index. CI, confidence interval; OR, odds ratio; SNP, single nucleotide polymorphism.

Following the MR analysis, the primary IVW method revealed a causal link between the relative abundance of seven genetically predicted bacterial taxa and FI, as illustrated in Figure 2 (*q <* 0.1). Comprehensive results are provided in Supplementary Table S4.

To elaborate, at the class level, *Bacteroidia* exhibited a protective effect against FI [odds ratio (OR) = 0.962, confidence interval (CI) = 0.928–0.997, *p* = 0.033]. Conversely, *Betaproteobacteria* was associated with an elevated risk of FI (OR = 1.050, CI = 1.002–1.101, *p* = 0.042). At the genus level, four gut microbiota entities were found to exert a positive causal influence on FI development. These included *Allisonella* (OR = 1.033, CI = 1.007–1.059, *p* = 0.012), *Bifidobacterium* (OR = 1.043, CI = 1.009–1.078, *p* = 0.013), *Clostridium innocuum group* (OR = 1.023, CI = 1.001–1.045, *p* = 0.036), and *Eubacterium coprostanoligenes group* (OR = 1.056, CI = 1.019–1.094, *p* = 0.003). Additionally, the *Eubacterium ruminantium group* (OR = 0.973, CI = 0.950–0.997, *p* = 0.028) exhibited a potentially negative causal effect on FI development. These results are graphically represented in scatter plots (Supplementary Figure S1) and forest plots, detailing the causal effects of gut microbiota on FI risk with individual SNPs (Supplementary Figure S2).

Notably, Cochrane’s *Q* test findings (Table 1) revealed a lack of significant heterogeneity among the selected SNPs (*p* > 0.05). Moreover, MR Egger tests for pleiotropy indicated no evidence of pleiotropic effects in our MR study (*p* > 0.05; Table 1). Although the leave-one-out method suggested that individual SNPs might introduce some bias in genetic prediction (Supplementary Figure S3), further MR-PRESSO analysis did not identify any significant outliers (all *p* > 0.05 for the global test). Importantly, MR-PRESSO analysis also confirmed the absence of horizontal pleiotropy (all *p* > 0.05).

**Table 1:** The sensitivity analyses of heterogeneity results and pleiotropy results associated with gut microbiota and FI.

Levels	Gut microbiota	MR-Egger		IVW		Egger intercept	SE	*p*-value
		*Q*	*p*-value	*Q*	*p*-value			
Class	*Bacteroidia*	13.734	0.248	13.774	0.315	0.001	0.003	0.861
Class	*Betaproteobacteria*	14.826	0.096	15.022	0.131	0.002	0.006	0.738
Genus	*Allisonella*	7.135	0.309	11.686	0.111	0.02	0.01	0.098
Genus	*Bifidobacterium*	5.776	0.834	7.055	0.795	−0.004	0.003	0.285
Genus	*Clostridium innocuum* group	3.171	0.869	6.718	0.567	−0.013	0.007	0.102
Genus	*Eubacterium coprostanoligenes* group	4.182	0.964	4.227	0.979	−0.001	0.005	0.835
Genus	*Eubacterium ruminantium* group	21.042	0.177	24.685	0.102	−0.007	0.004	0.116

FI, frailty index; MR, Mendelian randomization; SNP, single nucleotide polymorphism.

### 3.3 The result of reverse MR analysis

In the end, we performed reverse MR analyses to investigate potential associations between seven bacterial traits and frailty in the opposite direction. However, when using the IVW method, we did not find any statistically significant links between frailty and these seven bacterial traits. Specifically, for the *class Bacteroidia*, the odds ratio (OR) was 0.977 with a 95% confidence interval (CI) of 0.746 to 1.270, resulting in a *p*-value of 0.864. Similarly, for the *class Betaproteobacteria*, the OR was 1.137 (95% CI: 0.773, 1.672, *p* = 0.515). For the *genus Allisonella*, the OR was 0.957 (95% CI: 0.473, 1.936, *p* = 0.903), and for the *genus Bifidobacterium*, the OR was 1.143 (95% CI: 0.847, 1.544, *p* = 0.381). Additionally, for the *genus Clostridium innocuum group*, the OR was 1.096 (95% CI: 0.623, 1.927, *p* = 0.751), for the *genus Eubacterium coprostanoligenes group*, the OR was 1.052 (95% CI: 0.779, 1.420, *p* = 0.743), and for the *genus Eubacterium ruminantium group*, the OR was 0.911 (95% CI: 0.549, 1.398, *p* = 0.760). These results remained consistent across various sensitivity analyses, as outlined in Table 2.

**Table 2:** Effect estimates of the associations of FI with seven gut microbiota in the reverse MR analyses.

Levels	Gut microbiota	Methods	Number of SNPs	OR	95% CI	*p*-value	MR-Egger (intercept *p*-value)
Class	*Bacteroidia*	IVW	14	0.977	0.746–1.270	0.864	0.591
Class	*Betaproteobacteria*	IVW	14	1.137	0.773–1.672	0.515	0.412
Genus	*Allisonella*	IVW	14	0.957	0.473–1.936	0.903	0.823
Genus	*Bifidobacterium*	IVW	14	1.143	0.847–1.544	0.381	0.392
Genus	*Clostridium innocuum* group	IVW	14	1.096	0.623–1.927	0.751	0.098
Genus	*Eubacterium coprostanoligenes* group	IVW	14	1.052	0.779–1.420	0.743	0.534
Genus	*Eubacterium ruminantium* group	IVW	14	0.911	0.594–1.398	0.67	0.825

CI, confidence interval; FI, frailty index; MR, Mendelian randomization; OR, odds ratio; SNP, single nucleotide polymorphism; IVW, inverse variance weighted.

## 4 Discussion

This study represents the inaugural comprehensive, large-scale MR analysis examining the genetic basis for a causal relationship between gut microbiota and the FI. Prior research on this association predominantly utilized clinical trials and animal studies (Chen et al., 2022), which involved collecting and analyzing fecal samples from patients with frailty using 16S rRNA gene sequencing (Jackson et al., 2016; Margiotta et al., 2020; Lauretani et al., 2021). However, these approaches often faced challenges due to confounding factors such as age and smoking habits, complicating the establishment of a direct causal link. In contrast, our MR study provides robust evidence supporting a causal connection between specific gut microbiota compositions and an increased risk of FI. This pioneering work paves the way for identifying new biomarkers in future frailty research, marking a significant advancement in the field.

Among the prevalent bacterial phyla residing in the human gut microbiota, prominent members include *Proteobacteria*, *Verrucobacteria*, *Actinobacteria*, *Clostridium*, *Bacteroidetes*, and *Firmicutes*. Notably, *Bacteroidetes* and *Firmicutes* collectively constitute approximately 90% of the entire human gut microbiota composition (Rinninella et al., 2019). The ELDERMET cohort, established in 2007 and involving over 750 Irish individuals, stands as one of the most thoroughly studied populations regarding gut microbiota, shedding light on its complex relationship with health. This cohort highlighted the benefits of *Bacteroides*, *Alistipes*, and *Parabacteroides* in individuals over 65 compared to younger, healthy controls (Claesson et al., 2011). Further, a comprehensive study of over 9,000 individuals across various age groups revealed that *Bacteroides* remains prominent in those aged 85 and above, with a decrease in microbiota distinctiveness suggesting a potential link to lower survival rates within 4 years (Wilmanski et al., 2021). Conversely, a study on 32 sedentary women over 65 engaging in physical training showed that improvements in gait speed correlated with an increased abundance of *Bacteroides* (Morita et al., 2019). Additionally, research from the Heymans Elderly Center found a 6% reduction in *Bacteroides* among frail elderly individuals, highlighting a potential inverse causal relationship with frailty syndrome (van Tongeren et al., 2005). These findings collectively emphasize the critical role of Bacteroides in the development and understanding of frailty.

*Allisonella*, a member of the *Firmicutes* phylum, plays a unique role in the human gut microbiota through its ability to metabolize and produce histamine. Previous studies have linked a decreased *Firmicutes*/*Bacteroidetes* (F/B) ratio with frailty (Grigor’eva, 2020; Afonso et al., 2021), noting an inverse relationship between the predominant phyla, *Firmicutes* and *Bacteroidetes*. Imbalances in these phyla can lead to chronic inflammation (Lauretani et al., 2021). Additionally, *Firmicutes* can metabolize dietary compounds like choline, L-carnitine, and betaine into trimethylamine (TMA) (Yang et al., 2019), which is associated with frailty. Diets high in fats have been shown to negatively affect the gut microbiota, reducing *Bacteroidetes* levels while promoting the growth of Firmicutes, including opportunistic pathogens. Such dietary patterns increase intestinal mucosal permeability and the risk of systemic inflammation, potentially contributing to frailty (Murphy et al., 2015). Thus, the relative proportions of *Bacteroidetes* and *Firmicutes* are being explored as potential biomarkers for healthy aging, dietary habits, and lifestyle choices (Gorvitovskaia et al., 2016).

*Betaproteobacteria*, a class within the *Proteobacteria* phylum, has been found to vary in abundance between young adults and the elderly, with the latter group showing increased levels of *Actinomycetes*, particularly *Bifidobacterium*, and *Proteobacteria* (Salazar et al., 2017). However, it’s noteworthy that *Proteobacteria* can also contribute to frailty through its ability to metabolize dietary components into trimethylamine (TMA) (Yang et al., 2019). Additionally, aging is associated with a decline in *Firmicutes* and a rise in *Proteobacteria* and *Streptococcidae*, changes that are exacerbated by frailty and comorbidities (Biagi et al., 2010; Odamaki et al., 2016). Our study suggests a potential positive correlation between *Betaproteobacteria* levels and frailty risk, contrasting with the lack of a clear connection between overall *Proteobacteria* levels and frailty, highlighting the need for further research in this area.

The *Clostridium innocum group*, part of the *Clostridium* genus, consists of Gram-positive bacteria adapted to hypoxic or microaerobic conditions. An increase in *Clostridium* abundance correlates with higher levels of trimethylamine (TMA) (Chen et al., 2022), which may contribute to an increased risk of frailty. In a recent investigation by Lim et al. (2021), they examined the correlation between gut microbiota and frailty in a cohort of 176 elderly Korean individuals. Their findings revealed a significant increase in the abundance of the opportunistic pathogen *Clostridium* in elderly individuals afflicted with frailty. Furthermore, it’s noteworthy that the *Clostridium innocum group* exhibits a positive correlation with the incidence of colitis (Lunken et al., 2021). In summary, while this study signifies the initial identification of a plausible causal relationship between the *Clostridium innocum group* and frailty risk, further research is essential to elucidate the potential biological mechanisms linking them.

Rampelli et al. (2013) also noted an elevation in the levels of *Eubacterium* and *Bifidobacterium* within the elderly population. Moreover, an increase in species count from *Akkermansia*, *Bifidobacterium*, and *Christensenaceae* was observed in the gut microbiota of centenarians (aged 99–104 years) and semi-supercentenarians (aged 105–109 years) (Biagi et al., 2016). Given the age-related nature of frailty, our research suggests a potential positive causal link between *Bifidobacterium* and frailty. Conversely, frailty is associated with an overexpression of *Eubacterium* (Jackson et al., 2016; Maffei et al., 2017). Frail individuals exhibit a higher abundance of *Eubacterium dolichum* compared to their non-frail counterparts (Jackson et al., 2016). However, in the gut microbiota of Sardinian centenarians, there was a decline in *Eubacterium rectale* levels coupled with an increase in *Bifidobacterium adolescentis* (Wu et al., 2019). Notably, *Eubacterium* citrate and related strains, regarded as longevity markers, surged by approximately 15-fold among centenarians (Biagi et al., 2010). Nonetheless, to date, no reports have explored the connection between individuals with debilitating syndromes and the *Eubacterium ruminantium group* or the *Eubacterium coprostanogenes group*. In animal research, the *Eubacterium ruminantium group* has demonstrated the ability to fortify the intestinal barrier and reduce the body’s inflammatory response (Fan et al., 2023; Hu et al., 2023), potentially mitigating frailty occurrence, aligning with our study’s findings. Regarding the *Eubacterium coprostanogenes group*, existing research links its increase to a higher risk of colorectal adenoma (Zapico et al., 2022; Xiang et al., 2023), while its association with frailty warrants further investigation. In summary, substantial individual disparities exist among the elderly, intertwined with intricate relationships among gut microbiota, dietary habits, and geographical locations. These findings suggest that the composition and diversity of gut microbiota undergo age-related changes, impacting the efficiency of the immune system, a pivotal factor in averting age-related ailments (Hasan and Yang, 2019; Rinninella et al., 2019).

This study presents several significant strengths. Firstly, it marks the inaugural application of a two-sample MR analysis to explore potential causal links between gut microbiota and FI. In contrast to traditional observational studies, this approach significantly reduces the potential for bias resulting from confounding variables and the risk of reverse causality. Secondly, the utilization of summary-level data on gut microbiota is derived from the largest GWAS to date, incorporating data from multiple human populations. This robust dataset enhances the generalizability of our findings across diverse human groups. Furthermore, it is important to emphasize the substantial epidemiological implications of MR analysis. Its utility is poised for continued growth in the foreseeable future, given the expanding availability of genetic data and the ongoing development of innovative methodologies. MR analysis is positioned to remain an invaluable tool for unraveling the causal relationships between risk factors and disease outcomes.

Our study, while contributing valuable insights, is subject to certain limitations. Initially, the standard MR methodology employed assumes a linear relationship between exposure and outcome, thereby limiting our ability to detect any non-linear associations or threshold effects between gut microbiota and FI. Furthermore, our analysis was confined to bacterial taxa at the genus level, excluding more detailed investigations at the species or strain levels. Predominantly, the study’s cohort consisted of individuals of European descent, which may affect the applicability of our findings across varied ethnic backgrounds. Additionally, the significance level for selecting instrumental variables (IVs) related to gut microbiota was set beyond the usual genome-wide threshold, necessitating FDR correction to mitigate the risk of false positives. The relatively small sample size for gut microbiota also means that the reverse MR analysis might suffer from weak instrumental bias, and the possibility of a reverse causal relationship cannot be entirely ruled out. It’s also important to note the modest effects of the bacterial traits observed, and the absence of other independent GWAS of FI with adequate sample sizes for validation purposes. Our study also lacks experimental models to establish a direct causal link between gut microbiota and FI, which should be a focus for future research. Lastly, the lack of data on FI subtypes warrants further investigation as more comprehensive information becomes available.

## 5 Conclusion

In conclusion, we employed a Mendelian randomization (MR) approach to elucidate the potential causal relationships between gut microbiome composition and frailty index (FI), a vital indicator of health in the elderly. Our findings reveal a significant association between specific gut microbiota and the risk of frailty, suggesting a direct genetic influence on these associations. These results not only advance our understanding of the gut microbiome’s role in aging and frailty but also highlight the potential for genetic predispositions to shape microbiome composition, thereby influencing health outcomes in the elderly.

## Data availability statement

The original contributions presented in the study are included in the article/Supplementary material, further inquiries can be directed to the corresponding author.

## Ethics statement

All studies included in cited genome-wide association studies had been approved by a relevant review board, and participants gave informed consent.

## Author contributions

ZW: Methodology, Visualization, Writing – original draft, Writing – review & editing. SH: Data curation, Writing – original draft. YX: Visualization, Writing – original draft. YZ: Supervision, Writing – review & editing. YG: Writing – review & editing. JG: Funding acquisition, Resources, Supervision, Writing – review & editing. XL: Formal analysis, Writing – review & editing.

## Footnotes

^1^ https://www.r-project.org/
