# Supplementary material for: Genetically supported causality between gut microbiota and frailty: a two-sample Mendelian randomization study
Source: Front Microbiol. 2024 Apr 17;15:1324209. doi: 10.3389/fmicb.2024.1324209 (PMC11089315; doi:10.3389/fmicb.2024.1324209)
Supplement: Supplementary file 1 [file Table_1.DOCX]

**Supplementary Figures**

**Supplemental Figure 1.** The scatter plots the 3 MR approaches for association between gut microbiota and FI.

**Supplemental Figure 2.** The forest plots for the association between gut microbiota and FI.

**Supplemental Figure 3.** The leave-one-out sensitivity analysis for the association between gut microbiota and FI.

**Supplemental Figure 1. The scatter plots for association between gut microbiota and FI.**


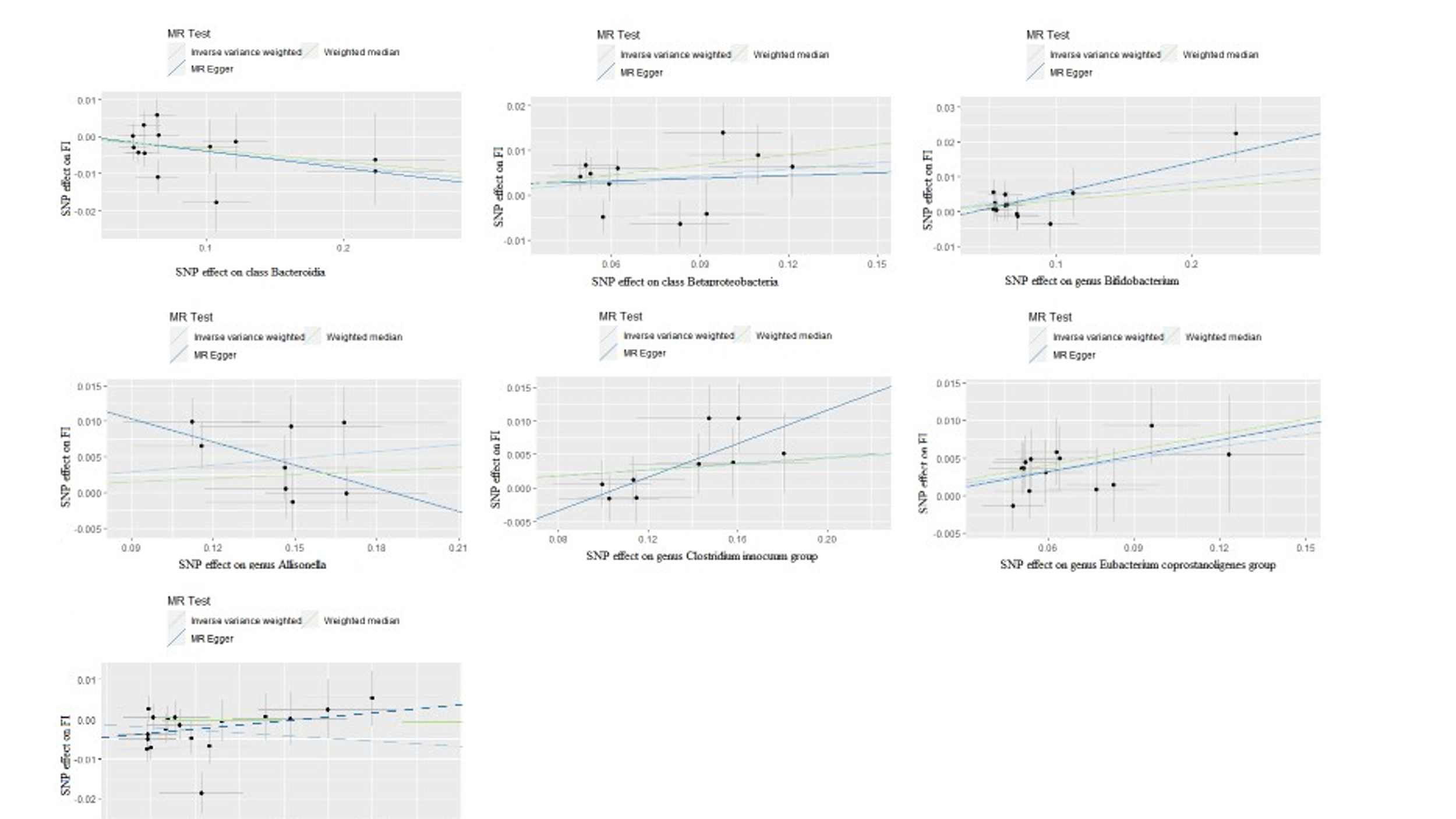


SNP effects were plotted into lines for the inverse-variance weighted test (light blue line), MR-Egger (blue line), weighted median (green line). The slope of the line corresponded to the causal estimation.

**Supplemental Figure 2. Forest plots for causal effects of gut microbiota on FI risk with individual SNPs.**


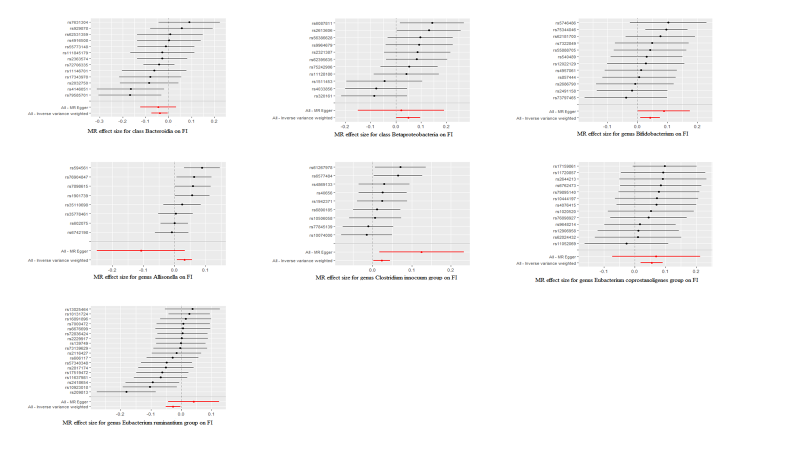


Black points denote the effect estimates of class *Bacteroidia*, class *Betaproteobacteria*, genus *Allisonella*, genus *Bifidobacterium* genus *Clostridium innocuum group,* genus *Eubacterium coprostanoligenes group* and genus *Eubacterium ruminantium group* on the risk of FI using single SNPs, and the black lines signify the 95% CIs of the estimates. The red points symbolize overall effect estimates of the gut microbiota using the Egger and IVW method, and the red lines indicate their 95% CIs**.**

**Supplemental Figure 3. Plots for "leave-one-out" analysis for causal effect of gut microbiota on FI risk.**

**Plots for "leave-one-out" analysis** **for causal effect of gut microbiota on FI risk.** Leave-one-out plot helps determine whether the overall effect is altered by one or more specific genetic variants by sequentially re-evaluating causal estimates after discarding one SNP at a time.The black points denote effect estimates of the gut microbiota after discarding a certain SNP, and black lines signify the corresponding 95% CIs of estimates. Red points symbolize overall causal effect estimate of the gut microbiota on FI risk using a set of SNPs, and red lines indicate the corresponding 95% CIs.
